# Exploring the Efficacy of Using *Geotrichum fermentans*, *Rhodotorula rubra*, *Kluyveromyce marxiamus*, Clay Minerals, and Walnut Nutshells for Mycotoxin Remediation

**DOI:** 10.3390/toxins16060281

**Published:** 2024-06-20

**Authors:** Gintarė Vaičiulienė, Jurgita Jovaišienė, Rimvydas Falkauskas, Algimantas Paškevičius, Neringa Sutkevičienė, Audronė Rekešiūtė, Šarūnė Sorkytė, Violeta Baliukonienė

**Affiliations:** 1 Animal Reproduction Laboratory, Large Animal Clinic, Veterinary Academy, Lithuanian University of Health Sciences, Tilzes Str. 18, LT-47181 Kaunas, Lithuania; neringa.sutkeviciene@lsmu.lt (N.S.); audrone.rekesiute@lsmu.lt (A.R.); sarune.sorkyte@lsmu.lt (Š.S.); 2 Department of Food Safety and Quality, Veterinary Academy, Lithuanian University of Health Sciences, Tilzes Str. 18, LT-47181 Kaunas, Lithuania; jurgita.jovaisiene@lsmu.lt (J.J.); violeta.baliukoniene@lsmu.lt (V.B.); 3 National Food and Veterinary Risk Assessment Institute, J. Kairiukscio Str. 10, LT-08411 Vilnius, Lithuania; rimvydas.falkauskas@nmvrvi.lt; 4 Laboratory of Biodeterioration Research, Institute of Botany, Nature Research Centre, Akademijos Str. 2, LT-08412 Vilnius, Lithuania; algimantas.paskevicius@gamtc.lt

**Keywords:** AFB1, ZEA, DON, T-2 toxin, in vitro, yeasts, polysaccharides, clay, walnut nutshells

## Abstract

The aim of this study was to evaluate the effectiveness of nine different biological compounds to reduce mycotoxins concentrations. The hypothesis of this study was that a static in vitro gastrointestinal tract model, as an initial screening tool, can be used to simulate the efficacy of *Geotrichum fermentans*, *Rhodotorula rubra*, *Kluyveromyce marxiamus* yeast cell walls and their polysaccharides, red and white clay minerals, and walnuts nutshells claiming to detoxify AFB1, ZEA, DON, and T-2 toxin mycotoxins. Mycotoxin concentrations were analyzed using high-performance liquid chromatography (HPLC) with fluorescent (FLD) and ultraviolet detectors (UV). The greatest effects on reducing mycotoxin concentrations were determined as follows: for AFB1, inserted *G. fermentans* cell wall polysaccharides and walnut nutshells; for ZEA, inserted *R. rubra* and *G. fermentans* cell walls and red clay minerals; for DON, *R. rubra* cell wall polysaccharides and red clay minerals; and for T-2 toxin, *R. rubra* cell walls, *K. marxianus,* and *G. fermentans* cell wall polysaccharides and walnut nutshells. The present study indicated that selected mycotoxin-detoxifying biological compounds can be used to decrease mycotoxin concentrations.

## 1. Introduction

Mycotoxins, a class of toxic secondary metabolites naturally produced by various mold species, pose a significant threat to human and animal health due to their ubiquity and potential for contamination in various food and feed supplies [1]. *Aspergillus*, *Penicillium*, *Fusarium*, and *Alternaria* microscopic species fungi are the main mycotoxins producers [2]. Nowadays, more than 400 potentially toxic mycotoxins produced by more than 100 species of fungi have been identified. Mycotoxins constitute a structurally diverse group of low-molecular-weight toxic compounds, which is generally less than 1000 Da, and mycotoxigenic mold growth is essential for mycotoxin production, but the presence of mold species does not indicate toxin production [3]. Contamination of feed with mycotoxins in the dairy sector can cause serious food and feed safety issues, as well as negative impacts and significant losses to the ruminant industry [4].

Dairy cattle are often exposed to mycotoxins because of the large proportion of maize silage in their ration every day [5]. In fact, maize silage is several times more prone to the contamination of mycotoxins compared to grassland products and can occur before, during, or after harvest [6,7]. Usually, ensiled forages, maize, and grass silages often contain multiple mycotoxins, and the main and most frequently detected mycotoxins are AFB1 (aflatoxin B1), DON (deoxynivalenol), ZEA (zearalenone), and the T-2 toxin [8]. Meanwhile, HT-2 toxin, enniatins (ENN), nivalenol (NIV), fumonisins (FUM), fumaric acid (FA), and beauvericin (BEA) mycotoxins are determined less often and in smaller doses [9]. Feed contaminated with these toxins can cause mycotoxicosis in dairy cattle, characterized by a variety of clinical signs depending on the toxin and its doses [10]. Mycotoxicosis is usually divided into two forms: acute mycotoxicosis resulting from a large single dose of mycotoxins and chronic mycotoxicosis due to the continuous consumption of low amounts of mycotoxins over time. The toxic effects of various mycotoxins are conditioned by their bioaccessibility, bioavailability, and metabolic fate. The bioavailability of mycotoxins depends on their digestive stability and release from food matrixes, while bioaccessibility refers to the capability of a toxic compound released from a food matrix to pass across the intestinal barrier [11]. Usually, in vitro static methods that simulate the gastrointestinal tract are widely applied to predict the bioaccessibility and bioavailability of various mycotoxins [12]. The toxic level of mycotoxins causing acute disease in dairy cattle is 100 µg/kg for AFB1, 400 µg/kg for ZEA, and more than 100 µg/kg for T-2 [13]. However, chronic aflatoxicosis, caused by small exposure to several mycotoxins over time, is a more common animal health problem. Generally, in cattle, mycotoxins can cause reduced feed intake, alter ruminal fermentation and feed utilization, reduce the growth rate, inhibit protein synthesis, milk production, intestinal barrier integrity, mucin production, and the immune system, and cause serious reproductive problems [14,15]. In comparison, ruminants may be less affected by certain mycotoxins compared to monogastric, which is attributed to microbial activity in the rumen, which can change the chemical structure of the mycotoxin into less toxic compounds [16]. Mycotoxins can be harmful to animal health, and specific toxic clinical signs appear depending on the individual mycotoxin, as shown in Table 1.

In many countries, mycotoxin concentrations in feed and their products are meticulously regulated by legislation [17]. The specific values of maximum residue limits (MRLs) can vary by country. However, the European Union regulation on feedstuffs has so far established aflatoxin concentrations (AFB1, AFB2, AFG1, and AFG2) by Directive 32/2002 (European Communities 2002). Moreover, additional “guidance values” have been published by the European Commission for several other compounds such as DON, ZEA, fumonisins (FUM), ochratoxin A (OTA) (European Commission 2006), and T-2 and HT-2 toxins (European Commission 2013) [1,7].

One of the key challenges in today’s development to prevent animal exposure to mycotoxins is to find effective and natural silage additives (mycotoxin detoxifiers). Usually, they can be divided into two main categories: adsorbing agents (mycotoxin binders) and bio-transforming agents (mycotoxin modifiers) [18]. So, each subcategory has its own mode of action: binders absorb mycotoxins in the gastrointestinal tract and prevent mycotoxin absorption, whereas mycotoxin modifiers transform mycotoxins of microorganisms/enzymes into non- or less-toxic metabolites [19]. One of the most used binders is clay minerals, and depending on their structure and physicochemical properties, they can absorb mycotoxins in a different way. However, each type of clay has its own specific mycotoxin binding capacity and efficiency adsorption depending on both the clay and mycotoxin properties [20]. In addition, there is increasing interest in new and innovative biological materials that would be ecological, sustainable, and safe for the environment. So, one of the promising current alternatives of mycotoxin binders is waste residual biomass such as shells of different nuts, such as walnut nutshells [21]. Among biosorbents, nut shells have several advantages over other materials because they are not perishable, contain a large number of polysaccharides, have high porosity, and are of no commercial value [22]. Also, various yeast species and polysaccharides extracted from yeast cell walls can be used as natural mycotoxin modifiers. The types of yeast used for mycotoxin detoxification are mainly the *Saccharomyces genus* and *Saccharomyces cerevisiae* species [23]. Yeasts and yeast cell walls showed that their β-D-glucans composition and tridimensional network can chemically adsorb mycotoxins and transform them into non- or less-toxic metabolites, thus reducing the absorption of mycotoxins in the small intestine, reducing the accumulation of mycotoxins in specific organs, increasing their clearance, and protecting vital organs from the effects of mycotoxins [24,25].

The hypothesis of this study was that *Geotrichum fermentans*, *Rhodotorula rubra*, *Kluyveromyce marxiamus*, clay minerals, and walnut nutshells can be used as an alternative to conventional means for the decontamination of mycotoxins. Biological compounds have been divided into two groups according to their mode of action: (a) mycotoxin modifiers (*Geotrichum fermentans*, *Rhodotorula rubra*, *Kluyveromyce marxiamus* yeasts, and their cell wall polysaccharides) and (b) mycotoxin binders (clay minerals and walnut nutshells).

The aim of this study was to evaluate the effectiveness of selected individual biological compounds for the decrease in AFB1, ZEA, DON, and T-2 toxin concentrations.

## 2. Results

### Analysis of Selected Biological Compounds Effect on the Mycotoxin’s Concentrations Reduction

**Figure 1:**
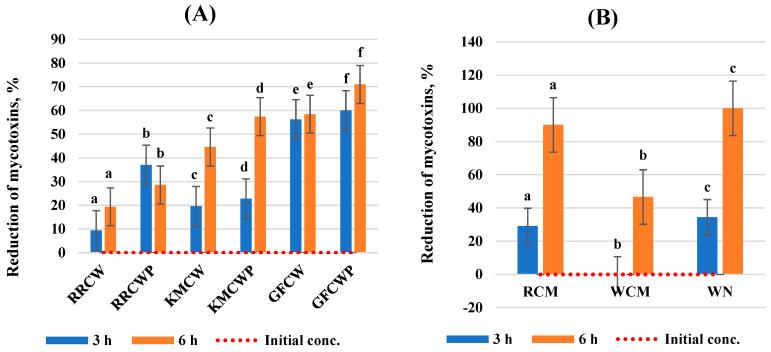
Reduction in aflatoxin B1 by biological compounds: (**A**) yeast cell walls and their polysaccharides; (**B**) mineral and biological absorbents. ^a–f^ Values within a column without a common superscript letter differ (*p* < 0.05). RRCW–*R. rubra* cell walls, RRCWP–*R. rubra* cell wall polysaccharides, KMCW–*K. marxianus* cell walls, KMCWP–*K. marxianus* cell wall polysaccharides, GFCW–*G. fermentans* cell walls, GFCWP–*G. fermentans* cell wall polysaccharides, RCM–red clay minerals, WCM–white clay minerals, WN–walnut nutshells.

**Figure 2:**
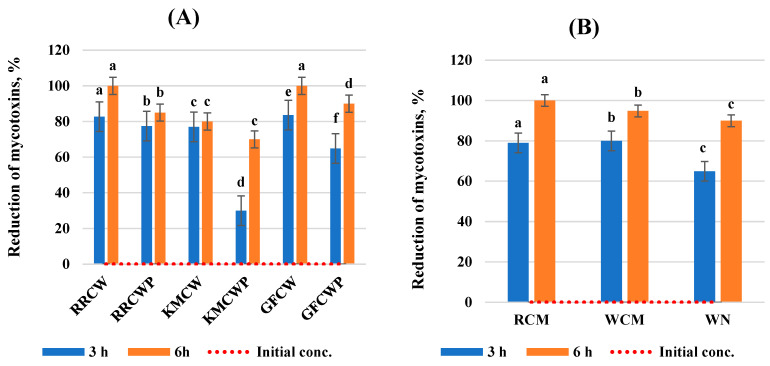
Reduction in zearalenone by biological compounds. (**A**) Yeast cell walls and their polysaccharides; (**B**) mineral and biological absorbents. ^a–f^ Values within a column without a common superscript letter differ (*p* < 0.05). RRCW–*R. rubra* cell walls, RRCWP–*R. rubra* cell wall polysaccharides, KMCW–*K. marxianus* cell walls, KMCWP–*K. marxianus* cell wall polysaccharides, GFCW–*G. fermentans* cell walls, GFCWP–*G. fermentans* cell wall polysaccharides, RCM–red clay minerals, WCM–white clay minerals, WN–walnut nutshells.

**Figure 3:**
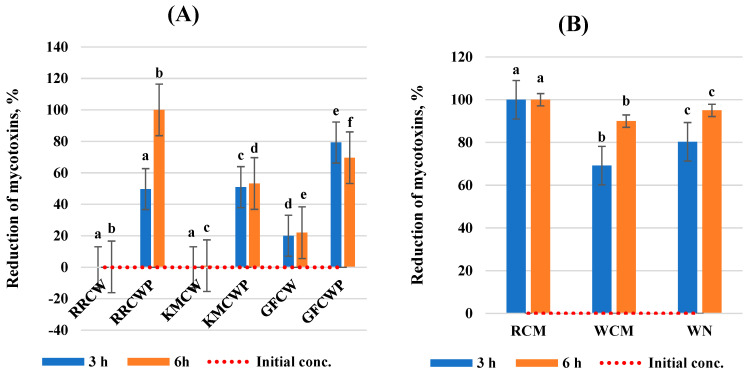
Reduction in deoxynivalenol by biological compounds. (**A**) Yeast cell walls and their polysaccharides; (**B**) mineral and biological absorbents. ^a–f^ Values within a column without a common superscript letter differ (*p* < 0.05). RRCW–*R. rubra* cell walls, RRCWP–*R. rubra* cell wall polysaccharides, KMCW–*K. marxianus* cell walls, KMCWP–*K. marxianus* cell wall polysaccharides, GFCW–*G. fermentans* cell walls, GFCWP–*G. fermentans* cell wall polysaccharides, RCM–red clay minerals, WCM–white clay minerals, WN–walnut nutshells.

**Figure 4:**
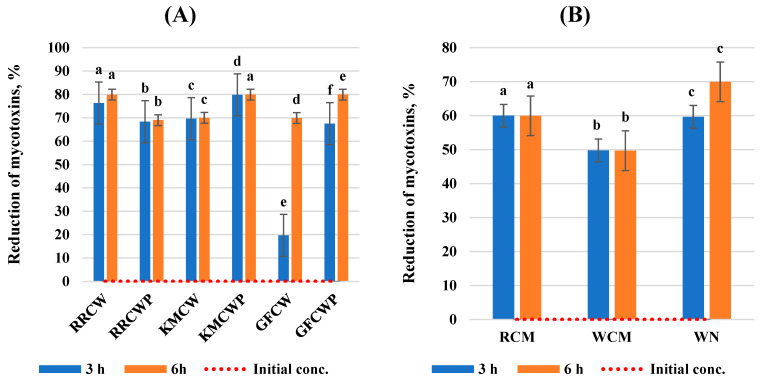
Reduction in T-2 toxin by biological compounds. (**A**) Yeast cell walls and their polysaccharides; (**B**) mineral and biological absorbents. ^a–f^ Values within a column without a common superscript letter differ (*p* < 0.05). RRCW–*R. rubra* cell walls, RRCWP–*R. rubra* cell wall polysaccharides, KMCW–*K. marxianus* cell walls, KMCWP–*K. marxianus* cell wall polysaccharides, GFCW–*G. fermentans* cell walls, GFCWP–*G. fermentans* cell wall polysaccharides, RCM–red clay minerals, WCM–white clay minerals, WN–walnut nutshells.

This work evaluated the effect of selected different biological compounds on the reduction in AFB1, ZEA, DON, and T-2 toxin mycotoxin concentrations in a static in vitro model of the gastrointestinal tract. The results of the analysis are expressed as the mycotoxin reduction at two incubation times and are summarized in Figure 1, Figure 2, Figure 3 and Figure 4. All tested individual biological compounds were grouped into two main groups: yeast cell walls and their polysaccharides (*n* = 6) and mineral and biological absorbents (*n* = 3).

While analyzing the effect of individual biological compounds on the reduction in the mycotoxins’ concentrations, all tested biological absorbents were able to bind to AFB1, and the binding efficacy of yeast cell walls and their polysaccharides varied from 9.4% to 71.0%, whereas mineral and biological absorbents varied from 29.1% to 100.0%.

The highest effect of yeast cell walls and their polysaccharides on AFB1 reduction was determined after 3 and 6 h of incubation by GFCW and GFCWP compared to other yeast cell walls and their polysaccharides. The lowest effect on mycotoxin reduction at both incubation times was determined with inserted RRCW. The highest efficiency to bind to AFB1 was found with inserted GFCWP (71.0%) after 6 h of incubation with a 15.51% higher efficacy compared to after 3 h of incubation (60.0%).

The sequestration rates of AFB1 by mineral and biological absorbents were higher using RCM and WN than those with WCM at both incubation times. RCM and WN were able to bind to AFB1 with the highest efficacy after 6 h of incubation (>90.05%).

After evaluating the effect of ZEA reduction, all tested biological compounds showed the highest efficiency after 6 h of incubation (>69.96%).

The best results for the reduction in ZEA by yeast cell wall and their polysaccharides after 3 and 6 h of incubation were obtained with inserted RRCW and GFCW, rather than with KMCWP, which showed the lowest reduction effect of all the tested compounds. RCW and GCW have the highest efficiency in binding to ZEA, which was equal to 100.0% after 6 h of incubation.

It was determined that the reduction in ZEA by mineral and biological absorbents was greater by RCM and WCM compared to WN at both incubation times. After 6 h of incubation, they were able to bind to ZEA with 18.34% higher efficacy (>94.0%) than after 3 h of incubation (>79.0%).

The reduction in DON by RRCWP and GFCWP was the highest (*p* < 0.05) of all tested yeast cell walls and their polysaccharides. After 6 h of incubation, they were able to bind to ZEA with a higher efficacy (>69.0%) compared to after 3 h of incubation (>49.0%). RRCWP has the highest efficiency able to bind to DON, which was equal to 100.0% after 6 h of incubation. However, RRCW, KMCW, and GFCWP did not effectively sequester DON.

All tested mineral and biological adsorbents were able to bind to DON with a very high efficacy (>69.18%). DON reduction was highest in RCM and equal to 100.0% after both incubation times (*p* < 0.05) compared to WCM and WN. However, WCM and WN were able to bind DON 30.82% and 19.68% after 3 h of incubation and 10.0% and 5.0% after 6 h of incubation, respectively.

The reduction in the T-2 toxin by yeast cell walls and their polysaccharides was highest with inserted RRCW and KMCWP (*p* < 0.05) compared to GFCW. RRCW and KMCWP obtained similar results after 3 and 6 h of incubation; they were able to bind to the T-2 toxin with a higher efficacy (>76.31%). The lowest reduction in mycotoxins was determined with inserted GFCW after 3 h of incubation.

While analyzing the mycotoxin reduction using mineral compounds, we determined very similar results for RCM and WCM at both incubation times; RCM was able to bind T-2 by more than 59.0%, while WCM was able to bind more than 49.0%. The best results of T-2 toxin reduction were determined with inserted WN after 6 h of incubation, which was able to bind to the T-2 toxin with a 14.71% higher efficacy (>69.0%) than after 3 h of incubation (>59.0%).

## 3. Discussion

The current study shows that *G. fermentans*, *R. rubra*, *K. marxiamus*, clay minerals, and walnut nutshells can reduce the concentrations of mycotoxins.

Recently, there has been an increase in the number of sustainable, innovative, and bio-acceptable materials that can be used instead of chemical fungicides to reduce the concentration of mycotoxins, thus improving dairy cattle feed quality [26,27]. The main goal of the use of biological compounds is to reduce the toxicity of a certain compound by absorbing or transforming it into less toxic compounds [28]. Thus, the main objective of our study was to select and investigate other innovative and biologically active substances such as yeasts and their polysaccharides, mineral compounds, and walnut nutshells. The obtained research results confirm that our investigated biological compounds have statistically reliable decontamination ability against various mycotoxins and can improve the quality of silage produced in Lithuania. Based on the literature, AFB1 is not considered a major problem in many European countries, but ZEA and DON have been identified increasingly and in larger quantities in ensiled feed [29].

So, we started our research with the assumption that if mycotoxin adsorption did not occur in vitro, it was highly unlikely that it would occur in vivo. In our study, a static gastrointestinal model in vitro has been successfully used as an initial screening tool to evaluate the efficacy of selected mycotoxin-detoxifying compounds in reducing mycotoxin concentrations, which is essential for simulating the physiological conditions of dairy cattle rumen [30]. To evaluate the mycotoxin concentration variations over time, the selected biological compounds were tested at two incubation times. Also, pH 6.8 was selected in the study to simulate physiological pH in the rumen of cattle.

The results of our research are the first study of its kind in Lithuania, where the effects of biological compounds on the reduction in mycotoxin concentrations are evaluated using a static model of the gastrointestinal tract in vitro. As there have not been many studies performed on this topic, it was very difficult to evaluate and interpret the obtained results. However, the results of this study showed an effective and reliable reduction in AFB1, ZEA, DON, and T-2 toxin concentrations in a static gastrointestinal model in vitro by using selected biological compounds and confirmed the results obtained by other authors. It should be noted that our research results indicate that selected biological compounds have different effects on individual mycotoxins and no single biological compound has been identified that would effectively reduce the concentrations of all tested mycotoxins at both incubation times.

Previous research has shown that yeast can be used to remove mycotoxins by using living cells, cell walls, or their polysaccharides. It was determined that yeasts and the structure of their polysaccharides are dynamic and can adapt to various physiological and morphological changes. Moreover, for mycotoxin binding, β-D-glucan and mannan oligosaccharide are responsible, which bind to mycotoxins via hydrogen bonding and van der Waal forces [31,32]. Also, previous studies analyzed the adsorption capacity of different yeasts against mycotoxins, but these studies mainly focused on *S. cerevisiae* yeast [33,34]. For this reason, in our research, we were searching for a new species of yeast, isolated from various substrates, that may have a similar effectiveness in reducing mycotoxins. So, it was found that *R. rubra*, *K. marxianus,* and *G. fermentans* can statistically and reliably (*p* < 0.05) reduce the concentrations of AFB1, ZEA, DON, and the T-2 toxin. Similar results were determined by Kawtharani et al. [35], who also established that a *Geotrichum candidum* strain could effectively reduce mycotoxin concentrations, especially the T-2 toxin. Malinee Intanoo et al. [36] found that the *K. marxianus* species can effectively detoxify AFB1 and AFM1, while according to Jakopović et al. [37], *K. marxianus* can be used to bind AFB1, OTA, and ZEA. According to our study results, the best results for AFB1 concentration reductions were established with inserted *G. fermentans* cell walls and their polysaccharides; the best results for ZEA were achieved with *R. rubra* cell walls and *G. fermentans* cell wall polysaccharides; the best results for DON were achieved with *R. rubra and G. fermentans* cell wall polysaccharides; and the best results for the T-2 toxin were achieved with *R. rubra* cell walls and *K. marxiamus* cell wall polysaccharides. The results we obtained were similar to Ran Xu et al.’s [38] research results, who also determined that yeast cell walls and their polysaccharides have high efficiency regarding binding to a wider range of mycotoxins such as AFB1, ZEA, DON, or OTA, thereby reducing the negative impact of mycotoxins.

Recently, many mycotoxin binders of different origins have been widely used. So, in the next phase of the experiment, we chose two mineral absorbents—red and white clay— as reliable materials to reduce the concentration of mycotoxins and a new, innovative, ecological, and waste-free bio absorbent—walnut nutshells. The most widespread class of mycotoxin adsorbents is aluminum silicate minerals due to their clear mechanism of action. As they are the most used and researched, they have been widely used to improve the quality of feed [39]. Currently, a promising alternative is biosorbents, which are obtained by processing various fruits or vegetables, because they promote waste-free consumption [40,41]. So, this was one of the first studies to use walnut nutshells to reduce the concentration of mycotoxin in Europe. Also, very little research has been performed on this topic worldwide. Walnut nutshells have several advantages over other biosorbents because they are widely available, have a high content of polysaccharides and great porosity, and are of no commercial value. A considerable amount of byproduct is formed when nuts are processed, of which the largest part is shells (67% of the total weight of the nuts), so these properties have led us to use nutshells as an ecological, effective, sustainable, innovative, and cost-effective biosorbent [42,43]. Our research results, which were similar to other authors’ results, confirmed that walnut nutshells can be effectively used to reduce the concentrations of mycotoxins, like other minerals. It was established that red clay minerals statistically and reliably reduce AFB1, ZEA, DON, and T-2 toxin (*p* < 0.05), while white clay minerals only had the same result for ZEA (*p* < 0.05) at both incubation times. Meanwhile, positive results were also found with walnut nutshells, as they statistically and reliably reduced AFB1, DON, and the T-2 toxin at both incubation times.

## 4. Conclusions

The results of our study showed the high efficacy of the selected biological compounds in reducing mycotoxin concentrations. A static in vitro model of the gastrointestinal tract was applied as an initial screening tool aiming to evaluate the efficacy of these compounds. This model allows for easy customization according to the products tested (e.g., yeasts and their cell wall polysaccharides, clay minerals, and walnut nutshells). This study revealed that all tested biological compounds absorbed AFB1, ZEA, DON, and the T-2 toxin to a certain extent, except the DON concentration by *Rhodotorula rubra* and *Kluyveromyce marxianus* cell walls after 3 h of incubation. The highest effect on the reduction in mycotoxin concentrations was determined with inserted *Geotrichum fermentans*, *Rhodotorula rubra,* and *Kluyveromyce marxiamus* cell walls and their polysaccharides, red clay minerals, and walnut nutshells (*p* < 0.05). Additionally, further detailed studies are necessary using different selected biological compound compositions to investigate their mechanisms of action.

## 5. Materials and Methods

### 5.1. Biological Materials

Three yeast strains isolated from various substrates (corn, vegetables, and fruits) grown in Lithuania, coded as *Kluyveromyces marxianus*, *Geotrichum fermentans*, and *Rhodotorula rubra*, were investigated. The strains were obtained from the Biodeterioration Research Laboratory culture collection belonging to the Institute of Botany of Nature Research Centre (Vilnius, Lithuania). All strains were maintained as active in YPD broth before the experiment and preserved at 4 °C.

Red clay (bentonite) and white clay (montmorillonite) were purchased from the company “Biocos” (Alsace, France). The clay minerals were 100% natural, raw, organic, naturally sun-dried, and mechanically ground into microparticles.

Walnut nutshells used in the experiments were collected in Lithuania, mechanically ground, and sieved to obtain particles with sizes between 1 and 2 mm. The nutshell material was washed abundantly with room-temperature water (22 °C) and dried in a muffle at 35 ± 2 °C for 48 h.

### 5.2. Preparation of Yeast Cell Wall Polysaccharides

Yeast cell wall polysaccharides were extracted from the yeast cell cultures maintained in YEP media using the glass bead breaking method (“micro method”) with some modifications. Cells were collected in the early exponential phase, considering that yeast cells were not exposed to limiting nutritional conditions during this growth phase. Primarily, yeast strain cells were propagated at 30 ± 2 °C for 2 days in falcon centrifuge tubes at normal oxygen conditions containing 10 mL of YPD broth (1% yeast extract, 2% peptone, and 2% glucose). The optical densities (ODs) of the samples were determined at 600 nm and adjusted to 2.0 with sterile distilled water using the Specord Plus UV/Vis Spectrophotometer (Analytik Jena, Jena, Germany). After incubation, monocultures of analyzed yeast strains were centrifuged at a relative centrifugal force (RCF) of 3468× *g* for 10 min (ECOspin III, Oberhausen, Germany) and the supernatants were washed three times with PBS buffer to remove any contents of residual culture medium. Yeast cell pellets were then disrupted in glass tubes with 0.5 mL of Tris-Cl 10 mm at pH 8 with 0.5 g of glass beads (0.45–0.55 mm in diameter) via mixing for four cycles of 1 min each at 1 min intervals on ice using a digital vortex mixer (Biosan Bio Vortex V1, Riga, Latvia). The cycles were stopped when more than 95% of cells were broken. Then, the surface yeast cell suspension was collected, and the glass beads were washed with 1 mL of cold Tris-Cl buffer. The collected supernatant and glass beads were centrifuged again under the same conditions. OD was determined at 600 nm and adjusted to 2.0 with sterile distilled water. The clear yeast cell wall polysaccharide supernatant was collected and stored at −20 °C until the experiment.

### 5.3. Preparation of the Static Gastrointestinal Model In Vitro

A static gastrointestinal model in vitro was created using a previous method with some modifications, as described by Keller et al. [44]. The gastric simulation solution was composed of saline and enzymes: 125 mM NaCl, 7 mM KCl, 45 mM NaHCO_3_, and 3 g/L pepsin (porcine gastric mucosa, 800–2500 V/mg) at pH 3. The spiking solution consisted of 5 µg/mL of AFB1, 500 µg/mL of ZEA, 5000 µg/mL of DON, and 250 µg/mL of the T-2 toxin, dissolved in an ethanol/H_2_O mixture (50/50, *v*/*v*). All reaction solutions were prepared immediately before the experiment. The biological compounds used in the experiment were divided into two main groups according to biological compound materials.

The following biological compounds were used:

1. RRCW—*Rhodotorula rubra* cell walls.
2. RRCWP—*Rhodotorula rubra* cell wall polysaccharides.
3. KMCW—*Kluyveromyce marxianus* cell walls.
4. KMCWP—*Kluyveromyce marxianus* cell wall polysaccharides.
5. GFCW—G*eotrichum fermentans* cell walls.
6. GFCWP—G*eotrichum fermentans* cell wall polysaccharides.
7. RCM—red clay minerals.
8. WCM—white clay minerals.
9. WN—walnut nutshells.

To prepare a static gastrointestinal tract model in vitro, in a 50 mL incubation flask, we mixed 2.5 mL of each selected biological compound, 40 µL of the spiking mycotoxin solution, and 25 mL of the gastric simulation solution. Throughout the experiment, the pH of all reaction solutions was 6.8, adjustable as needed with 6 M of HCl (to simulate the physiological pH of the rumen). All samples throughout the experiment were incubated in a shaking incubator (150 rpm) in triplicate at 39 ± 2 °C. Then, solutions were centrifuged at 10,000× *g* for 10 min and 0 h, 3 h, and 6 h. Less than 1 mL of the collected supernatants was used for AFB1, ZEA, DON, and T-2 toxin determination via HPLC-FLD and HPLC-UV methodologies.

### 5.4. Determination of Mycotoxin Concentrations

The concentrations of AFB1, ZEA, and the T-2 toxin were tested using high-performance liquid chromatography (HPLC) with a fluorescent detector (FLD) (Model LCMS-8060 Shimadzu Corporation, Kyoto, Japan), and the concentration of DON was determined using HPLC with an ultraviolet detector (UV) (Model Sciex API 5000, McKinley Scientific, Sparta Township, NJ, USA). Samples were extracted in distilled water for DON, in methanol–water (75:25 *v*/*v*) for AFB1 and ZEA, and in methanol–water (60:40 *v*/*v*) for the T-2 toxin at constant mixing on a mechanical shaker (Phoenix Instrument RS-OS 20, Inc., Garbsen, Germany) for 60 min at 23 °C. After extraction, the samples were centrifuged at a relative centrifugal force (RCF) of 3468× *g* for 10 min (Centrifuge MPW-251, MPW, Warsaw, Poland). Later, the supernatants were filtered using PTFE syringe filters with pore diameters of 0.22 µm (Millex-GS, Millipore, Billerica, MA, USA) and diluted in phosphate-buffered saline (PBS). In the sample purification step, the extracts were passed through a multi-mycotoxin immunoaffinity column 11 + Myco MS-PREP^®^ (R-Biopharm AG, Darmstadt, Germany) according to the manufacturer’s recommendations. The prepared samples were subjected to high-performance liquid chromatography analysis, the parameters of which are given in Table 2. Chromatographic separation of mycotoxins was performed using a LiChrospher^®^ 100 RP-18 (Merck KGaA, Darmstadt, Germany), LiChroCART 250–4 column (250 mm × 4.0 mm, 5 µm; Supelco Park, Bellefonte, PA, USA). Mycotoxin concentrations were determined by comparing the maximum retention times using standard solutions. Mycotoxin concentrations were determined by correlating the peak area of the samples with the standard curves obtained using HPLC analysis of standard solutions.

### 5.5. Statistical Analysis

Statistical data analysis was performed using the qualitative analysis package IBM Statistics SPSS, version no. 25 (IBM, Chicago, IL, USA). To evaluate the effect of selected biological compounds on AFB1, ZEA, DON, and T-2 toxin mycotoxin concentrations, the data were analyzed using descriptive statistics and a one-way ANOVA. The differences in the test properties of the compared groups were expressed as mean values and the standard error of the mean (SEM), and differences between the compared groups were assessed using Fisher’s LSD test (α = 5%). The obtained results were statistically significant when *p* < 0.05.

## Figures and Tables

**Table 1:** Toxic effect of mycotoxins in dairy cattle.

Effect	AFB1	ZEA	DON	T-2/HT-2 Toxin	FUM	OTA
Carcinogenicity	√				√	√
Immunotoxicity	√	√	√	√		√
Hepatotoxicity	√	√		√	√	√
Nephrotoxicity				√		√
Neurotoxicity				√		
Teratogenicity						√
Dermal toxicity				√		
Gastrointestinal system toxicity			√			
Reproductive system toxicity		√		√		

**Table 2:** HPLC analysis parameters for mycotoxin detection.

Parameters	Mycotoxins			
	AFB1	DON	T-2 Toxin	ZEA
Column temperature	30 °C	30 °C	40 °C	30 °C
Mobile phase	H_2_O/ACN/MeOH (60:20:30)	H_2_O/ACN/MeOH (94:3:3)	H_2_O/ACN (40:60)	H_2_O/ACN/MeOH (46:46:8)
Fluorescent detector, wavelength λ (nm) (excitation and emission)	365 and 435	-	381 and 470	274 and 418
UV detector λ (nm)	-	218	-	-
Flow rate (mL/min)	1	1	1	1
Injection volume (μL)	100	100	100	100
Limit of detection (LOD) (μg/kg)	0.2	20	1.4	3

UV—ultraviolet rays; H_2_O—water; ACN—Acetonitrile; MeOH—Methanol.

## Data Availability

The data presented in this study are available within the article.

## References

[1] Orlov A.V., Znoyko S.L., Malkerov J.A., Skirda A.M., Novichikhin D.O., Rakitina A.S., Zaitseva Z.G., Nikitin P.I. (2024). Quantitative Rapid Magnetic Immunoassay for Sensitive Toxin Detection in Food: Non-Covalent Functionalization of Nanolabels vs. Covalent Immobilization. Toxins.

[2] Zhang D., Zhao L., Chen Y., Gao H., Hua Y., Yuan X., Yang H. (2022). Mycotoxins in maize silage from China in 2019. Toxins.

[3] Janik E., Niemcewicz M., Ceremuga M., Stela M., Saluk-Bijak J., Siadkowski A. (2020). Molecular Aspects of Mycotoxins—A Serious Problem for Human Health. Int. J. Mol. Sci..

[4] Gbashi S., Madala N.E., De Saeger S., De Boevre M., Adekoya I., Adebo O.A., Njobeh P.B., Njobeh P.B., Stepman F. (2018). The Socio-Economic Impact of Mycotoxin Contamination in Africa. Mycotoxins-Impact and Management, Strategies.

[5] Fink-Gremmels J. (2008). The role of mycotoxins in the health and performance of dairy cows. Vet. J..

[6] Zachariasova M., Dzuman Z., Veprikova Z., Hajkova K., Jiru M., Vaclavikova M., Zachariasova A., Pospichalova M., Florian M., Hajslova J. (2014). Occurrence of multiple mycotoxins in European feedingstuffs, assessment of dietary intake by farm animals. Anim. Feed Sci. Technol..

[7] Valgaeren B., Théron L., Croubels S., Devreese M., De Baere S., Van Pamel E., Daeseleire E., De Boevre M., De Saeger S., Vidal A. (2018). The role of roughage provision on the absorption and disposition of the mycotoxin deoxynivalenol and its acetylated derivatives in calves: From field observations to toxicokinetics. Arch. Toxicol..

[8] Wambacq E., Vanhoutte I., Audenaert K., Gelder L.D., Haesaert G. (2016). Occurrence, prevention and remediation oftoxigenic fungi and mycotoxins in silage: A review. J. Sci. Food Agric..

[9] Tangni E.K., Pussemier L., Van Hove F. (2013). Mycotoxin contaminating maize and grass silages for dairy cattle feeding: Current state and challenges. J. Anim. Sci. Adv..

[10] Chebutia Kemboi D., Antonissen G., Ochieng P.E., Croubels S., Okoth S., Kangethe E.K., Faas J., Lindahl J.F., Gathumbi J.K. (2020). A review of the impact of mycotoxins on dairy cattle health: Challenges for food safety and dairy production in sub-saharan sfrica. Toxins.

[11] Martínez-Alonso C., Taroncher M., Rodríguez-Carrasco Y., Ruiz M.J. (2023). Evaluation of the Bioaccessible Fraction of T-2 Toxin from Cereals and Its Effect on the Viability of Caco-2 Cells Exposed to Tyrosol. Toxins.

[12] González-Arias C.A., Marín S., Sanchis V., Ramos A.J. (2013). Mycotoxin bioaccessibility/absorption assessment using in vitro digestion models: A review. World Mycotoxin J..

[13] Whitlow L., Hagler W. (2008). Mold and Mycotoxin Issues in Dairy Cattle: Effects, Prevention and Treatment. Adv. Dairy Technol..

[14] Gonçalves B., Corassin C., Oliveira C. (2015). Mycotoxicoses in Dairy Cattle: A Review. Asian J. Anim. Vet. Adv..

[15] Reisinger N., Schurer-Waldheim S., Mayer E., Debevere S., Antonisse G., Sulyok M., Nagl V. (2020). Mycotoxin Occurrence in Maize Silage—A Neglected Risk for Bovine Gut Health?. Toxins.

[16] Rodriguez-Blanco M., Ramos A.J., Sanchis V., Marin S. (2021). Mycotoxins occurrence and fungal populations in different types of silages for dairy cows in Spain. Fungal Biol..

[17] Upadhaya S.D., Sung H.G., Lee C.H., Lee S.Y., Kim S.W., Cho K.J., Ha J.K. (2009). Comparative Study on the Aflatoxin B1 Degradation Ability of Rumen Fluid from Holstein Steers and Korean Native Goats. J. Vet. Sci..

[18] Panasiuk L., Jedziniak P., Pietruszka K., Piatkowska M., Bocian L. (2019). Frequency and levels of regulated and emerging mycotoxins in silage in Poland. Mycotoxin Res..

[19] Jard G., Liboz T., Mathieu F., Guyonvarch A., Lebrihi A. (2011). Review of mycotoxin reduction in food and feed: From prevention in the field to detoxification by adsorption or transformation. Food Addit. Contam. Part A Chem. Anal. Control Expo. Risk Assess..

[20] Kolosova A., Stroka J. (2011). Substances for reduction of the contamination of feed by mycotoxins: A review. World Mycotoxin J..

[21] Nadziakiewicza M., Kehoe S., Micek P. (2019). Physico-chemical properties of clay minerals and their use as a health promoting feed additive. Animals.

[22] Papadaki M.I., Mendoza-Castillo D.I., Reynel-Avila H.E., Bonilla-Petriciolet A., Georgopoulos S. (2021). Nut shells as adsorbents of pollutants: Research and perspectives. Front. Chem. Sci. Eng..

[23] Nahle S., Khoury A.E., Savvaidis I., Chokr A., Louka N., Atoui A. (2022). Detoxifcation approaches of mycotoxins by microorganisms, bioflms and enzymes. Int. J. Food Contam..

[24] Kim S.W., Holanda D.M., Gao X., Park I., Yiannikouris A. (2019). Effect of Naturally Co-Occurring Mycotoxins Contaminating Feed Ingredients Fed to Young Pigs: Impact on Gut Health, Microbiome, and Growth. Food Control.

[25] Habschied K., Krstanovic V., Zdunic Z., Babiic J., Mastanjević K., Šaric G.K. (2021). Mycotoxins Biocontrol Methods for Healthier Crops and Stored Products. J. Fungi.

[26] Ben Taheur F., Kouidhi B., Al Qurashi Y.M.A., Ben Salah-Abbès J., Chaieb K. (2019). Review: Biotechnology of mycotoxins detoxification using microorganisms and enzymes. Toxicon.

[27] Abdallah M.F., De Boevre M., Landschoot S., De Saeger S., Haesaert G., Audenaert K. (2018). Fungal Endophytes Control Fusarium graminearum and Reduce Trichothecenes and Zearalenone in Maize. Toxins.

[28] Gallucci M.N., Oliva M., Casero C., Dambolena J., Luna A., Zygadlo J. (2009). Antimicrobial combined action of terpenes against the food-borne microorganisms Escherichia coli, Staphylococcus aureus and Bacillus cereus. Flavour Fragr. J..

[29] Boudergue C., Burel C., Dragacci S., Favrot M.C., Fremy J.M., Massimi C., Prigent P., Debongnie P., Pussemier L., Boudra H. (2009). Review of mycotoxin-detoxifying agents used as feed additives: Mode of action, cacy and feed/food safety. EFSA Support. Publ..

[30] Yiannikouris A., André G., Buléon A., Jeminet G., Canet I., François J. (2004). Comprehensive conformational study of key interactions involved in zearalenone complexation with β-d-Glucans. Biomacromolecules.

[31] Yiannikouris A., André G., Poughon L., François J., Dussap C.G., Jeminet G. (2006). Chemical and conformational study of the interactions involved in mycotoxin Ccmplexation with β-d-Glucans. Biomacromolecules.

[32] Aguilar-Uscanga B., François J.M. (2003). A study of the yeast cell wall composition and structure in response to growth conditions and mode of cultivation. Lett. Appl. Microbiol..

[33] Hsu P.H., Chiang P.C., Liu C.H., Chang Y.W. (2015). Characterization of cell wall proteins in Saccharomyces cerevisiae clinical isolates elucidates Hsp150p in virulence. PLoS ONE.

[34] Piotrowska M., Masek A. (2015). Saccharomyces cerevisiae cell wall components as tools for ochratoxin a decontamination. Toxins.

[35] Kawtharani K., Beaufort S., Anson P., Taillandier P., Mathieu F., Snini S.P. (2022). Impact of the Inoculation Method of Geotrichum candidum, Used as Biocontrol Agent, on T-2 Toxin Produced by Fusarium sporotrichioides and F. langsethiae during the Malting Process. Toxins.

[36] Intanoo M., Kongkeitkajorn M.B., Suriyasathaporn W., Phasuk Y., Bernard J.K., Pattarajinda V. (2020). Effect of Supplemental Kluyveromyces marxianus and Pichia kudriavzevii on Aflatoxin M1 Excretion in Milk of Lactating Dairy Cows. Animals.

[37] Jakopovic Ž., Čiča K.H., Mrvčic J., Pucic I., Čanak I., Frece J. (2018). Properties and Fermentation Activity of Industrial Yeasts Saccharomyces cerevisiae, S. uvarum, Candida utilis and Kluyveromyces marxianus Exposed to AFB1, OTA and ZEA. Food Technol. Biotechnol..

[38] Xu R., Kiarie E.G., Yiannikouris A., Sun L., Karrow N.A. (2022). Nutritional impact of mycotoxins in food animal production and strategies for mitigation. J. Anim. Sci. Biotechnol..

[39] Liu M., Zhao L., Gong G., Zhang L., Shi L., Dai J. (2022). Invited review: Remediation strategies for mycotoxin control in feed. J. Anim. Sci. Biotechnol..

[40] Adamovic M., Stojanovic M., Grubišic M., Ileš D., Milojkovic J. (2011). Importance of aluminosilicate minerals in safe food production. Maced. J. Anim. Sci..

[41] Kabak B., Dobson A., Var I. (2006). Strategies to prevent mycotoxin contamination of food and animal feed: A review. Crit. Rev. Food Sci. Nutr..

[42] Pan Z., Zhang R., Zicari S. (2019). Integrated Processing Technologies for Food and Agricultural By-Products.

[43] Feizi M., Jalali M. (2015). Removal of heavy metals from aqueous solutions using sunflower, potato, canola and walnut shell residues. J. Taiwan Inst. Chem. Eng..

[44] Keller L., Abrunhosa L., Keller K., Rosa C.A., Cavaglieri L., Venancio A. (2015). Zearalenone and Its Derivatives α-Zearalenol and β-Zearalenol Decontamination by *Saccharomyces cerevisiae* Strains Isolated from Bovine Forage. Toxins.

